# The Dutch Auditory & Image Vocabulary Test (DAIVT): A New Dutch Receptive Vocabulary Test for Students

**DOI:** 10.5334/pb.552

**Published:** 2021-01-19

**Authors:** Ibrich Bousard, Marc Brysbaert

**Affiliations:** 1 Department of Experimental Psychology, Ghent University, Ghent, Belgium

**Keywords:** Dutch vocabulary, word knowledge, vocabulary test, receptive vocabulary, spoken word comprehension, individual differences

## Abstract

We introduce a new Dutch receptive vocabulary test, the Dutch auditory & image vocabulary test (DAIVT). The test is multiple choice and assesses vocabulary knowledge for spoken words. The measure has an online (available at *https://tpsurvey.ugent.be/limesurvey315/index.php/923234?lang=nl*) format, has free access, and allows easy data collection. The test was developed with the intent to enable testing for research purposes with university students. This paper describes the test construction. We cover three phases: 1) collecting stimulus materials and developing the test’s first version, 2) an exploratory item-analysis on the first draft (n = 93), and 3) validating the test (both the second and the final version) by comparing it to two existing tests (n = 270, n = 157). The results indicate that the test is reliable and correlates well with existing Dutch receptive vocabulary tests (convergent validity). The final version of the DAIVT comprises 90 test items and 1 practice item. It can be used freely for research purposes.

## INDIVIDUAL DIFFERENCES IN VOCABULARY KNOWLEDGE

Estimating individual differences in vocabulary knowledge is a key element in psychological research. Although word acquisition and word retrieval are complex constructs (ultimately requiring the development of detailed mathematical and computational models), much research about the impact of vocabulary knowledge can be done with rather simple tests estimating a person’s vocabulary size. To give some examples, better vocabulary knowledge has been linked to faster lexical processing (Mainz, Shao, Brysbaert, & Meyer, 2017), better listening comprehension (Andringa, Olsthoorn, van Beuningen, Schoonen, & Hulstijn, 2012), better top-down restoration of degraded speech (Benard, Mensink, & Başkent, 2014), and higher word production speed in verbal tasks (Rodríguez-Aranda & Jakobsen, 2011). Vocabulary knowledge is also the core index of crystallized intelligence (Horn & Cattell, 1967; Raven, 1948).

Vocabulary knowledge can be tested in many ways (Schmitt, Nation, & Kremmel, 2020; Webb & Nation, 2017). Some tests simply require participants to indicate which words they know from a list (e.g., Meara & Buxton, 1987). Other tests require participants to select the correct meaning among multiple alternatives (e.g., Raven, 1948). Still other tests stress the importance of word production instead of word perception and require participants to produce the correct word to a given definition or to a picture (e.g., Mainz et al., 2017). A further distinction can be made between vocabulary size (how many words a person knows) and vocabulary depth (how well a person knows each word). Finally, tests can present stimuli in spoken form or in written form.

Although it is tempting to search for the “best” format (indeed, much discussion has been devoted to this issue), from the perspective of test theory a much better approach is to make a distinction between manifest variables and latent variables. Manifest variables are the variables we can measure (i.e., the vocabulary tests we have at our disposal). Latent variables are the theoretical constructs we try to assess (i.e., vocabulary knowledge). Each manifest variable is an imperfect measure of the latent variable and in addition may load on other latent variables (e.g., reading skills, learning motivation, general intelligence, …). The best way to assess latent variables is to use the so-called multitrait-multimethod approach (Campbell & Fiske, 1959). In this method, each latent variable (trait) is measured with several tests, and researchers accept that latent variables do not exist in a vacuum but co-occur with other latent variables. A carefully selected range of tests can address multiple traits (latent variables) in parallel and estimate each trait properly by including several converging manifest variables. Evidence for the existence of a distinct latent variable for vocabulary knowledge comes from the observation that the various vocabulary test formats correlate well with each other (Mainz et al., 2017; Miralpeix & Muñoz, 2018; Schmitt, 2014; Vermeer, 2001).

Even within each test format it is good to have more than one test. Tests not only measure latent variables but are also affected by test-specific factors. Three of these factors are range effects, stimulus features, and experimenter bias. As for the first factor, a test only works if the items show good variability in the sample tested. Specifically related to vocabulary testing, a vocabulary test developed for the general population may not work well for university students, if all students know the words (e.g., Brysbaert, Sui, Dirix, & Hintz, 2020). Similarly, a test developed for children is unlikely to work well with adults, and the other way around, because adults know many more words than children. With respect to stimulus features, a test may contain suboptimal elements, such as outdated stimuli or unclear drawings. As for experimenter bias, a test may sample some word types more than other because it comprises only a small number of all possible words. Authors may vary in the words they include, which favor some participants. The best way to counter test-specific factors is to use tests from different groups of authors, so that convergent validity can be established.

For the above reasons, researchers ideally have access to a good variety of tests, both in terms of test formats and in terms of tests developed by different teams. In the discussion below, we focus on Dutch tests for receptive knowledge to be used with university students. Such tests are important as much psychological research is based on a convenience sample of university students.

## DUTCH RECEPTIVE VOCABULARY MEASURES

The majority of receptive vocabulary tests use a multiple choice test format. The most popular Dutch test to investigate individual differences in receptive vocabulary size is the Peabody Picture Vocabulary Test-III-NL (PPVT-III-NL; Schlichting, 2005). It measures spoken single-word comprehension. Participants hear a word and have to indicate which of four drawings agrees with the word. The test is untimed, individually administered, and norm-referenced. The latter makes use for clinical purposes possible. The test format does not require reading, as it involves auditory input and a pictorial representation of target and distractor words. Therefore the PPVT allows testing with participants of young age or participants with low reading skills.

Other researchers developed written multiple choice tests to estimate individuals’ Dutch receptive vocabulary size. Andringa et al. (2012) developed a written receptive vocabulary test for use with students in higher education. It consists of 60 items with five response alternatives, the last always being “I really don’t know”. Vander Beken, Woumans, and Brysbaert (2018) developed another multiple choice receptive vocabulary test for the same population. It consists of 75 items with four response alternatives, all presented in written form.^1^

A final format of receptive vocabulary test is the yes/no word decision task (Meara & Buxton, 1987). Participants get strings of letters and have to indicate whether they form existing words or are made-up nonwords. The format is often used in research because it allows rapid testing and is available for several languages (e.g., Amenta, Badan, & Brysbaert, 2020; Brysbaert, 2013; Lemhöfer & Broersma, 2012).

Mainz et al. (2017) presented seven different Dutch vocabulary tests, including both receptive and productive tests: (1) the Andringa et al. test (receptive, multiple choice), (2) Peabody (receptive, multiple choice), (3) a definition task (writing the correct word to a definition), (4) antonym multiple choice (receptive, choose the antonym of a target word among alternatives), (5) antonym generation (productive, writing the antonym), (6) synonym multiple choice, and (7) synonym generation. The authors found correlations ranging from r = .2 to r = .7 between the various tests and all tests loaded highly on a single factor (latent variable). The production tests loaded higher on the latent variable for university students, whereas the receptive tests did better for vocational high school students. The Peabody Picture Vocabulary Test did well in both groups and is the focus of the present study.

## THE PEABODY PICTURE VOCABULARY TEST (PPVT)

The original American version of the PPVT was introduced by Lloyd M. Dunn in 1959 (Dunn, 1959). Since then, four more American versions have been released, the PPVT-Revised (Dunn & Dunn, 1981), the PPVT-III (Dunn & Dunn, 1997), the PPVT-4 (Dunn & Dunn, 2007), and the PPVT-5 (Dunn, 2018). Norms and illustrations were revised for each version. The earliest versions (PPVT and PPVT-Revised) had a restricted age range focusing on children, but the latest versions allow test taking at all ages (from toddlers to 90-year-old adults). All American versions consist of two parallel forms, an A and a B form.

Because of the positive test results and advantages of the test format, a Dutch version of the PPVT was developed. Test items from both the A and B form of the American PPVT-III (Dunn & Dunn, 1997) were used as base material to develop the Dutch PPVT-III-NL (Schlichting, 2005). Many test items were not good enough to include in the Dutch version for various reasons (e.g., translation difficulties, no cultural relevance, outdated figures).

The PPVT-III-NL is administered individually and requires the presence of an examiner. No reading is required. Depending on the examinee’s performance, the test generally takes 10–15 minutes to complete. The examinee sits in front of a test folder and is offered one test item at a time. Each item consists of a set of four pictures, numbered from 1 to 4, including one target and three distractors. The pictures in the PPVT-III-NL are black line drawings on a yellow background. For each item, the examiner offers a spoken target word. Part of speech for the target words varies (noun, adjective, or verb). The examinee is asked to indicate the picture that best represents the word, by pointing to or by saying the number of the respective picture. The Dutch PPVT test was normed in 2004 and released in 2005. As of yet, no updated version of the Dutch PPVT has been published.

The order of test items is arranged according to increasing difficulty and the items are classified in sets of 12. There are 17 sets of 12 items in total. The order and number of administered sets depends on the examinee’s performance. The test is set up in such a way that examinees with a poorer Dutch receptive vocabulary complete fewer sets. The advantages of the test format, such as limited reading requirements and practicality of data collection, encouraged other researchers to develop English alternatives with a similar format for specific target groups (e.g., Anthony & Nation, 2017; Puimège & Peters, 2019).

## MOTIVATIONS FOR DEVELOPING A NEW TEST

Our primary incentive to develop a new test was the lack of a freely available alternative for the PPVT to be used in research with young adult advanced users of Dutch (typically university students). The PPVT-III-NL is a popular instrument in research to assess Dutch receptive vocabulary size (e.g., Benard et al., 2014; Jongman, Khoe, & Hintz, 2020; Mainz et al., 2017; Simonis, Van der Linden, Galand, Hiligsmann, & Szmalec, 2020) and existing research suggests that it performs well for this population. Mainz et al. (2017), for instance, found that the students’ PPVT-scores were successful predictors for performance (speed and accuracy) on a lexical processing task, with higher vocabulary scores being associated with better performance.

An extra test of the PPVT format is desirable for several reasons. First, some of the PPVT properties interfere with easy test administration. The PPVT does not provide an online version and demands the presence of an examiner to administer the test. The latter prevents researchers from testing more than one participant at a time. The PPVT test scores must be calculated manually, which is also time intensive. Some researchers avoided these problems by making their own online version of the PPVT-III-NL (Jongman et al., 2020; Mainz et al., 2017), but because the test is copyright protected it cannot be shared with other researchers.

A second limitation of the PPVT-III-NL is that it has a rather high cost, making it inaccessible for many (young) researchers. The test comes with a test folder, score forms, and a manual, which must be bought. This hinders extensive use of the test. A contrast can be made with the freely available yes/no Dutch test (Lemhöfer & Broersma, 2012). Everybody can run the test on their computers or implement new versions of it. As a result, the test has rapidly become standard in Dutch psycholinguistic research, even though it is too easy to make fine distinctions between high-level native speakers (Vander Beken et al., 2018).

A final limitation of the PPVT is that the answer format makes it difficult to construct alternative versions. The PPVT is based on line drawings, which require very specific (expensive) skills. Few people can make clear and appealing drawings, and the time cost involved reduces the number of alternatives that can be produced. Therefore, several researchers have tried out the use of photographs instead of line drawings (e.g., Anthony & Nation, 2017; Puimège & Peters, 2019). There is a much higher availability of free (standardized) photo databases compared to line drawing databases. This provides every researcher with the opportunity to include a wide range of target and distractor images. In addition, properties such as texture, shading, color, and other surface details of color photographs allow an easier representation and contextualization of target words. It is difficult to make clear line drawings for words like ‘calamity’ or ‘precarious’, whereas one can search for photographs depicting such situations. Photos also work better for objects requiring fine visual detail for recognition (e.g., it is very difficult to draw a mattress that everyone can name). For these reasons, line drawings are increasingly replaced by photographs in picture naming studies (e.g., Brodeur, Guérard, & Bouras, 2014).

To sum up, we wanted to develop an online Dutch receptive vocabulary test with pre-recorded audio stimuli and appealing photographs as targets and distractors, specifically directed to higher education students, so that reliable differences in vocabulary size can be determined for this group of participants. As Brysbaert et al. (2020) argued, the reliability of a test depends on the group for which it has been developed. Many general tests are of little use with university students, because most students perform at ceiling level. Similarly, tests developed for university students often have limited use outside higher education, because they are too difficult. Another concern we wanted to address, is practicality. We wanted to secure free access and easy availability for both researchers and participants, so that the test can be integrated seamlessly in psycholinguistic experiments.

## PRESENT STUDY

The present study describes and validated the Dutch Auditory & Image Vocabulary Test (DAIVT) for students in higher education. This involved three stages. In the first stage we selected 109 items with the same format as in the PPVT. On each trial a spoken word is presented together with four images: one target image and three distractor images. Participants have to select the target image. In phase two we tested the newly compiled test and ran a first item analysis. This allowed us to improve the test by taking into account the correlations of the items with the total scores. More specifically, some distractor and/or target images were replaced and some items were left out. In the third and final phase the DAIVT was validated by comparing performance on it with performance on two existing, good receptive vocabulary tests: the Dutch Peabody picture vocabulary test (PPVT-III-NL) and the receptive multiple choice test of Andringa et al. (2012). The new test was adapted two more times in the process. The final version of the DAIVT contains 90 items: 53 nouns, 20 adjectives, and 17 verbs (see Appendix A).

## PHASE I – COMPILING THE FIRST VERSION OF THE TEST MATERIALS

### PRELIMINARY ANALYSIS

We intended to make a test with a format similar to the PPVT, so the PPVT was used as a reference to develop the new test. Before starting the test construction for this project, the target words and respective target and distractor pictures of two PPVT tests were analyzed extensively. We used the standard American English Peabody Picture Vocabulary Test – 4 (PPVT-4-EN; Dunn & Dunn, 2007) and the Dutch Peabody Picture Vocabulary Test – III (PPVT-III-NL; Schlichting, 2005). All sets that can be administered from the age of 16 on were examined.

Data from The Dutch Lexicon Project 2 (DLP2) was used to obtain several values for the target words. The DLP2 offers lexical decision data for almost 30.000 Dutch words in the form of a list (Brysbaert, Stevens, Mandera, & Keuleers, 2016). This list also includes values per lemma for variables such as frequency (from SUBTLEX: word frequencies based on Dutch subtitles; Keuleers, Brysbaert, & New, 2010), word prevalence (word knowledge in the population), age of acquisition (AoA), and concreteness for the word. We were particularly interested in all these values, so that we could have an idea of the required difficulty of the words. Furthermore, we examined which images were used as distractors. This gave us a general idea of what words to use and how to select distractor images.

### WORD SELECTION FOR TARGETS AND DISTRACTORS

The primary objective was to compile a list with target words for the new test. All words would eventually need a representative photo. Thus, we selected a generous number of words, anticipating a decrease in the image selection process.

The most decisive selection criteria were frequency and prevalence. All words in the DLP2 list were ordered from high prevalence to low prevalence. We started selecting words at the top of the list and went gradually down. The goal was to avoid extremely difficult or very easy words, as these do not contribute much when looking at individual differences. Moreover, we wanted to develop a test that includes different word types. Therefore, the new target word list contained nouns, adjectives, and verbs. The words represented objects, actions, sceneries, and more abstract words. Words from different semantic categories were selected.

Once the list of target words was compiled, we looked for distractor words. The distractors usually belonged to the same semantic category as the target (e.g., aquatic animals, herbs, instruments, professions), had a perceptual match (e.g., same color, similar shape), or required a similar action (e.g., making gestures). As a rule of thumb, we tried to find distractors with slightly higher prevalence and frequency than target words. Items are unlikely to work well if the distractors of a well-known target word are unfamiliar words (e.g., the distractors tourniquet, semaphore, and stator for the target word “knife”) or if the distractors of a difficult word are familiar words (e.g., the distractors knife, fork, and flower for the target “tourniquet”). Authors of vocabulary tests further advise to make distractors slightly easier than target words, so that the main focus is on knowledge of the target word.

### IMAGE SELECTION

On the basis of the word list, we searched for high quality, ecologically valid photographs. Target and distractor images were collected by searching in several freely available and copyright free picture databases. As we depended on the availability of high quality photos, we had to drop several item candidates and sometimes change a word for which we could not find a good stimulus.

The Bank of Standardized Stimuli (BOSS) was used as a first important source to access high quality color photos from a wide range of categories (Brodeur et al., 2014). All pictures are a photographed object or living thing and are placed against a white background. Currently, the BOSS project offers a selection of 1468 normative pictures, normalized for dimensions such as name agreement, familiarity, visual complexity, and object agreement. Given the presence of these norms, all photos from the BOSS were considered appropriate to be included in the new test.

Moreno-Martínez and Montoro (2012) compiled a picture database with 360 colored photos against a white background, subdivided into 23 categories. The database offers photos normalized for the variables familiarity, name agreement, and visual complexity, among other things. The Moreno-Martínez and Montoro picture database was used supplementary to the BOSS database.

Neither of the previously mentioned picture databases included pictures related to humans or human acts (except for human body parts), nor pictures for words that require a more abstract representation, more context-specific information, more background information or more perspective. Therefore, many words we wished to include in the DAIVT exceeded the scope of the available normed databases. This required us to search for pictures on online copyright free and free-to-use photo websites, mainly Pixabay and Pxhere. Keywords, both in English and Dutch, were used to find the desired images.

**Figure 1:**
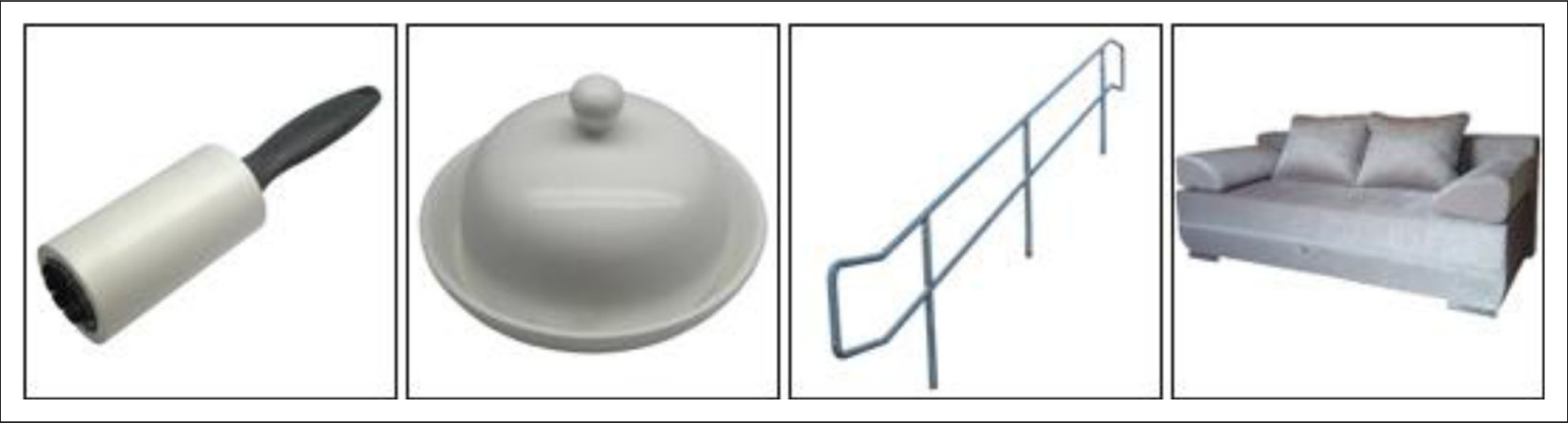
Target and distractor images for 1 test item; similar color scheme.

The image selection procedure was an extensive part of test construction. Compiling the pictures for the quartets required special attention. Images had to be clear and describe a specific word, also when shown in a row of four pictures on a rather small screen (e.g., of a smartphone). We made an effort to include photos that were not overly complex. For instance, we used a white background if possible and we tried to use the same color scheme for all pictures in an item (***Figure 1***). This was done primarily for nouns, representing objects. If one image had a white background, all other images from the same item had a white background too. Furthermore, actions, objects, or sceneries were zoomed in as much as we thought necessary and/or possible.

**Figure 2:**
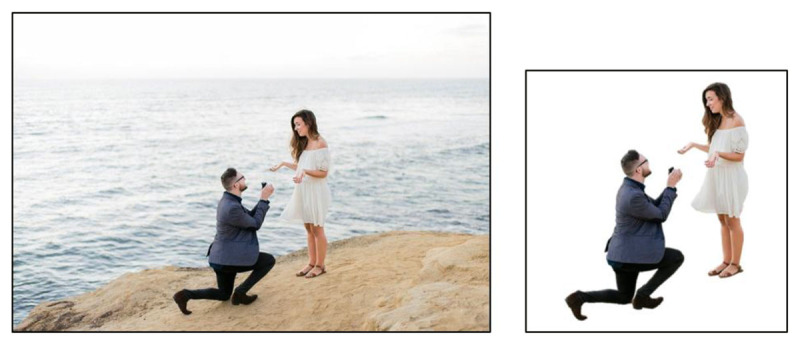
Source: Pixabay. Left image: original photo – right image: cropped, background removed (included in the DAIVT).

**Figure 3:**
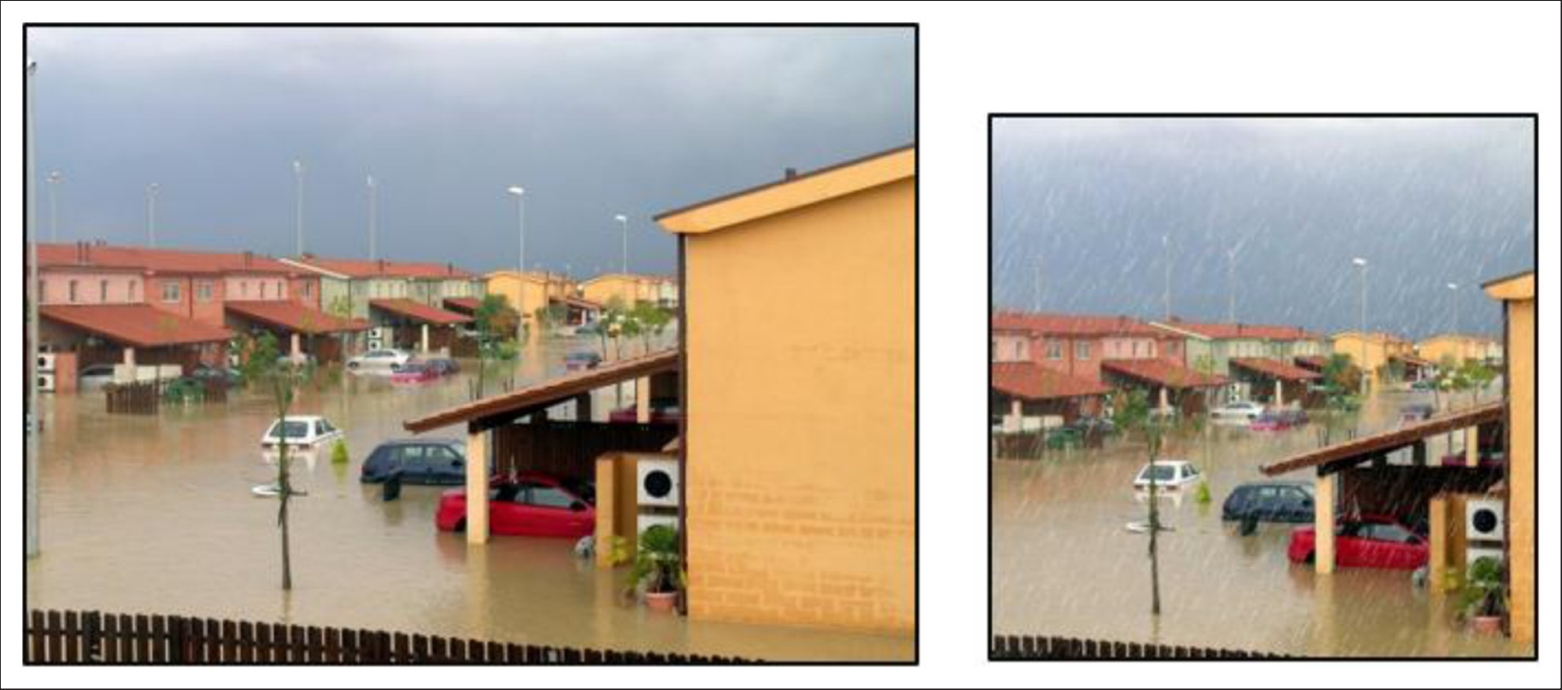
Source: Pixabay. Left image: original photo – right image: cropped, added rain filter (included in the DAIVT).

All pictures were cropped into a square picture ratio. Many pictures needed adaptation. The adjustments reduced visual complexity (e.g., adding a white background: ***Figure 2***) or added value to the test item (e.g., by adding a rain filter to a picture representing a flooded street for the target word *calamiteit* [calamity]; ***Figure 3***). Lastly, images for words that required a representation not yet available in a database were constructed separately (e.g., for abstract words such as *degressie* [degression] and *concentrisch* [concentric]). Candidate words for which no suitable images were found or could be made, were removed from the list. The first image selection procedure yielded 107 complete test items (64 nouns, 23 adjectives, 20 verbs).

In the end, more than 95% of target photos had distractor images that represented the same broad semantic category. To give an example, whenever the target picture depicted an inanimate object, the other pictures also showed inanimate objects. The same was true for animate beings, actions, sceneries, and more abstract representations. This also helped to reduce visual complexity of the four item pictures.

There was no separate image validation procedure, in which we tried to elicit the most likely name for each image, as it was not important for participants to name each and every picture correctly. It was only important that they selected the correct picture for a given spoken word.

### APPARATUS

Several web-based survey platforms were considered for designing and running the new test. We eventually settled for Limesurvey, which could handle the memory requirements for the test (given that we use picture materials) and which is made available by Ghent University for its researchers.

**Figure 4:**
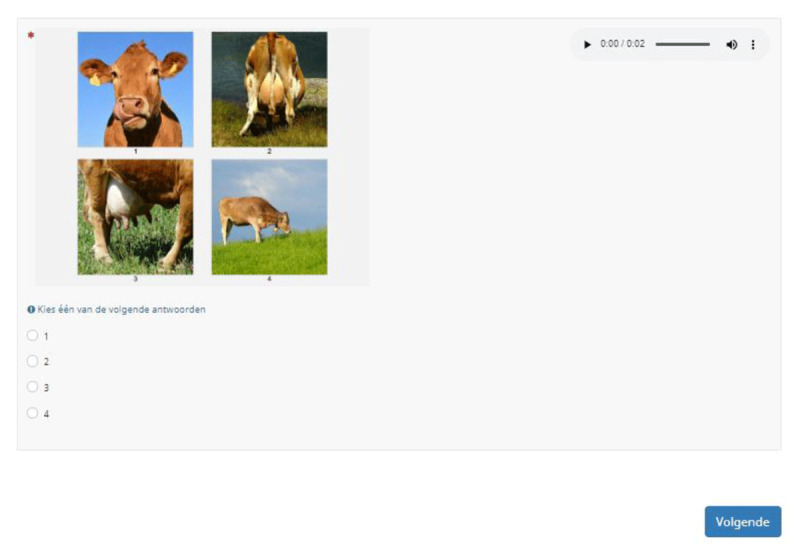
First item set-up (for the word ‘posterior’): a sound clip with the option to replay the target word, a collage with 1 target image and 3 distractor images (numbered from 1 to 4), and 4 radio buttons.

**Figure 5:**
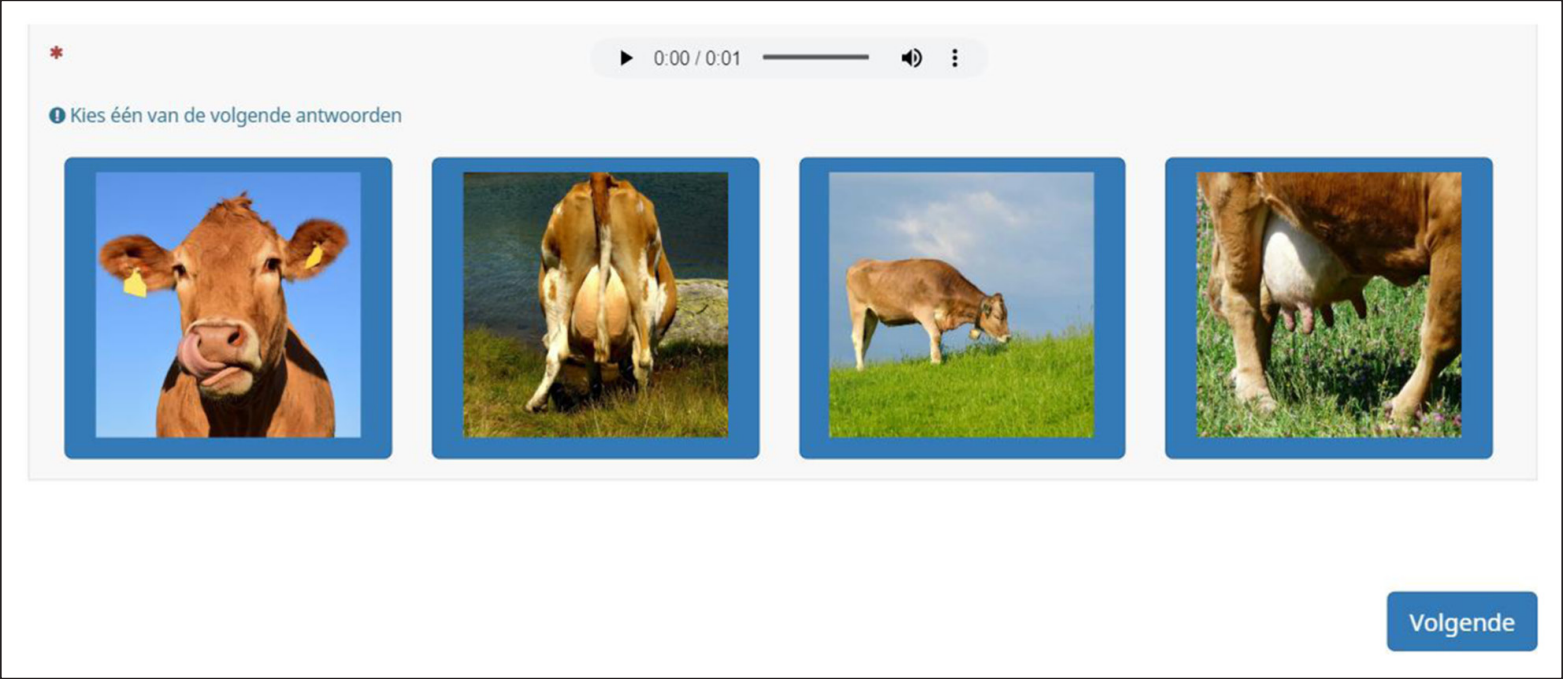
The DAIVT’s current item set-up: a sound clip with the option to replay the target word and 4 answer alternatives (1 target image, 3 distractor images) presented alongside each other. Participants click on the target picture.

All target words were recorded with a native Dutch (Flemish) female voice and stored as an audio clip. In a first version of the test, the four images on the screen were presented in a 2×2 array and numbered from 1 to 4. The answer buttons were placed separately under the array (***Figure 4***). However, upon testing it became clear that the full stimulus was not visible on most devices (e.g., laptops or smartphones), requiring a lot of scrolling by the participants. In addition, users suggested it would be more practical if they could click on the images directly. Therefore, we adapted the outline in such a way that the four images were presented alongside each other and could be selected by simply clicking on them. Limesurvey allowed this set-up. In this way we also eliminated the need for numbers underneath the images. ***Figure 5*** displays the selected item set-up. Test-takers are asked to select the photo they think best represents the spoken target word. The target word is played automatically once. Test-takers can replay the audio if wanted by clicking on the sound clip. By pressing on *Volgende* [Next], test-takers are directed to the next test item. No time limit is included, but participants have to select an image before they can move on to the next item (i.e., there is no possibility to skip unknown items).

## PHASE II – ITEM-ANALYSIS OF THE FIRST DRAFT

The next step in the test construction process was to look at the internal consistency and quality of the individual test items. Although the test’s target population was university students, we tested the first version in a wide population, as a wide range of performance is good for initial item evaluation. Therefore we sought to include participants regardless of age and educational level.

### METHOD

#### Participants

A link to the test was sent out via mailing lists and social media. No age restriction was set. A total of 93 people completed the test on a voluntary basis (37 male, 54 female, 2 not indicated). Ages varied between 17 and 65+ (*M = 35, SD = 13.5*). Participants had a varying education level. However, about 80% of participants owned a professional/academic bachelor’s degree or master’s degree.

#### Procedure

Participants completed the first version of the DAIVT with 107 test items and 2 practice items (*stoel* and *lezen*, [chair and reading] respectively). As previously explained, the test was run through Limesurvey. Participants accessed the online test via a link. The instructions included an informed consent and participants were instructed to indicate the image they thought best represented the spoken word. We advised them to use headphones or earphones. They were shown their total score at the end of the test as a reward for taking part.

#### Analysis

We performed a principal component analysis (PCA) with one component, as we wanted to address a single latent variable: vocabulary knowledge. This allowed us to discover the loadings and the uniqueness scores of the items. The loadings represent the correlations between the measured test items and the component. An item was considered good if it had a minimum factor loading of .20. Test items with a loading of .20 and below needed to be revised.

### RESULTS

Both practice items were answered correctly by all participants, indicating a sufficient understanding of the test instructions.

Regarding the test items, all 93 participants selected the correct target image for the target words *baret* and *afgepeigerd*. So, these items were too easy to be included. The average total score was 84 out of 107 (range 53–102, *SD = 11.6*). Although the test had a good reliability (Cronbach’s α = 0.90), the item-analysis indicated a factor loading below .20 for 20 items and 3 items loaded negatively with the total scale. On the positive side, 53 items had a factor loading of .30 or more.

### DISCUSSION AND TEST ADJUSTMENTS

The results from the first sample gave us a first insight into the test’s performance. In this phase, the goal was to do an item-analysis and a reliability analysis (Cronbach’s α). Both analyses reflected promising results. However, we needed to take into account that we intended to use the test in a student population, typically proficient users of Dutch. Because no age restriction was used in the first sample, the average age was relatively high (± 35 y/o). We expected student samples to be more homogenous, resulting in less variation in scores.

To improve the items that required revision, we looked at the individual responses. This allowed us to discover ambiguities in the test items. We concluded that some target photos did not sufficiently represent the target word or that some distractor photos could be confounded with the target image. The respective target and/or distractor images were adapted. This was the case, for instance, for the target word “aalscholver” [cormorant]. In the first version, all distractors were birds and this proved to be too difficult a task. So, we changed the distractors to other animals, one of which was “aal” [eel]. The same was true for the target “hellebaard” [halberd]. Participants were unable to select the correct image among other medieval weapons. So, we made the task easier by using a wider range of distractors. Again, we used the picture databases and free-to-use photo websites to find substitute images.

For a few items we discovered that the target word could be interpreted as referring to two pictures. As a result, participants knowing the word were not guaranteed a correct response. For these items, we replaced the ambiguous distractor by an unambiguous one.

Three test items, *sublimatie, animositeit*, and *kavalierperspectief*, were removed from the test, because we failed to find adequate photos for the target word or the word was too technical. Two target words, namely *abdominaal* and *pergola*, were changed to *pectoraal* and *prieel*, which previously were distractor images for the test item. The former target words were modified because the original test items had factor loadings lower than .20 and .0. *Pectoraal* and *prieel* were considered more favorable in terms of frequency and prevalence to fit in the test (Brysbaert et al., 2016).

In sum, the first item-analysis enabled us to make some necessary adjustments to the test. After this phase, the DAIVT comprised 104 test items.

## PHASE III – VALIDITY (PART 1)

The subsequent part of the test construction was to look at the DAIVT’s validity. The objective was to compare the results of the DAIVT with those of two existing good receptive vocabulary tests: Schlichting’s (2005) PPVT-III-NL and Andringa et al.’s (2012) receptive multiple choice test. Therefore, we tested new participants and administered the DAIVT with both other tests or with the PPVT-III-NL only (depending on time constraints).

### PEABODY PICTURE VOCABULARY TEST – III – NL (PPVT-III-NL) (SCHLICHTING, 2005)

**Figure 6:**
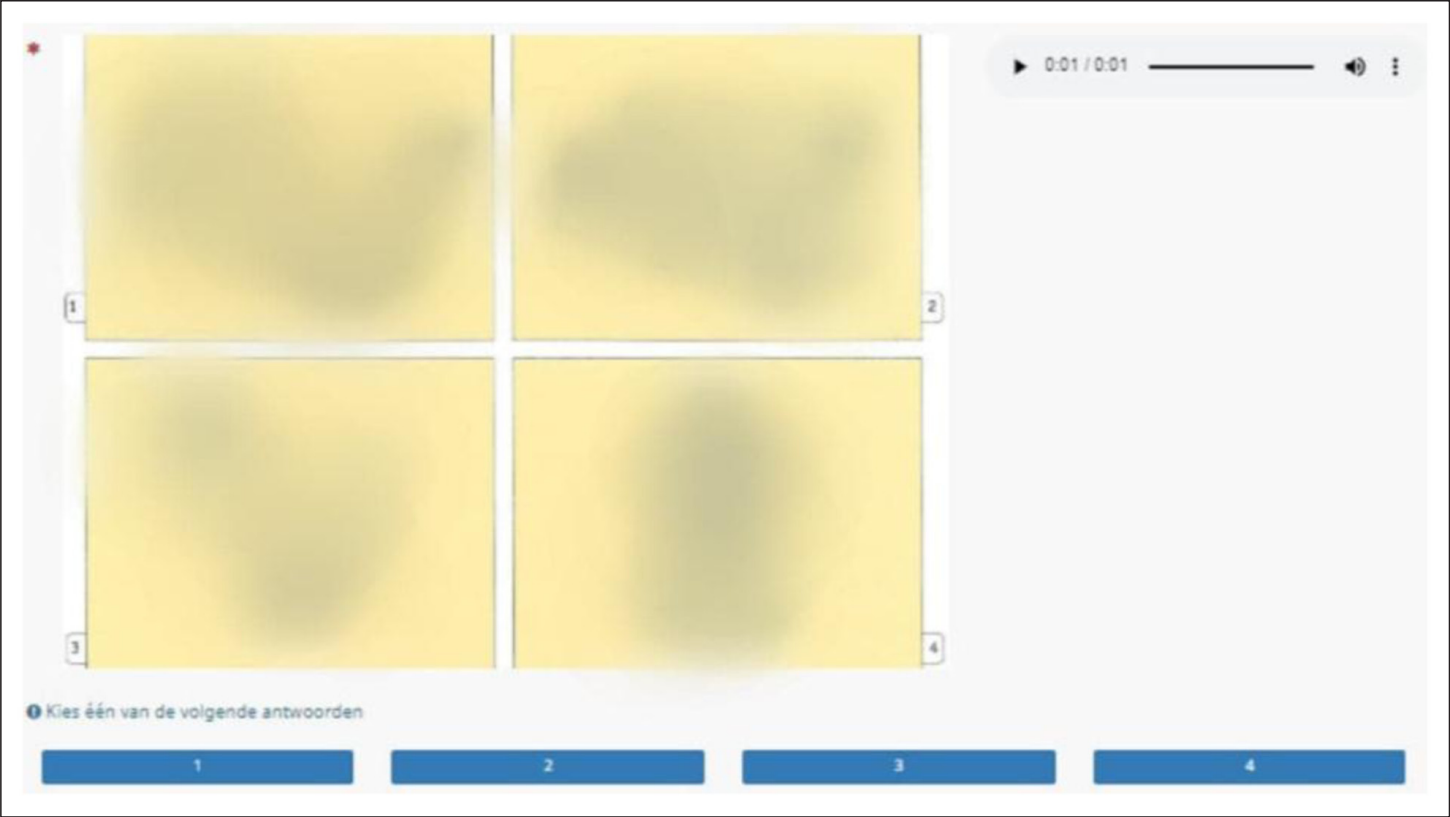
PPVT-III-NL (Schlichting, 2005); example item set-up (pictures are blurred because the test is copyright protected).

Naturally, we wanted to include the PPVT-III-NL to assess the DAIVT’s convergent validity. As mentioned in the introduction, the test usually demands the use of a test folder and an examiner. However, we made an online version for the present study. We added the test to the DAIVT questionnaire in Limesurvey. The target words were recorded with the same Flemish female voice. The PPVT administration rules were added to Limesurvey. The entry set of 12 items depended on the participants’ age. If more than four errors were made, previous sets were administered until the participant made fewer than four errors in a set. The ceiling set was the one in which the participant made nine or more mistakes. At that moment the test was stopped, unless a base set was not yet determined. A set was always administered in its entirety. ***Figure 6*** displays the PPVT item set-up in the Limesurvey questionnaire.

The PPVT-III-NL enables calculating a raw score, a standard score (*WBQ* or *woordbegripquotiënt* [word comprehension score]), a confidence interval, and a percentile. The raw score is calculated by subtracting the number of errors from the number of the ceiling item. The other mentioned values depend on the raw score and the examinee’s age and allow comparing participants between and within age groups. *WBQ* tables with different age categories were available based on a standardization sample for the test. The tables allow conversion from a raw score to a *WBQ* (normally distributed, *M = 100, SD = 15*). We calculated the *WBQ* for all participants that were tested in the present study. However, note that we will consistently use participants’ raw scores for all analyses described in this paper, as this is the score most comparable to the DAIVT score.

### RECEPTIVE MULTIPLE CHOICE TEST (ANDRINGA ET AL., 2012)

We chose to include Andringa et al.’s receptive multiple choice test for its practicality and performance in previous research. Mainz et al. (2017) recommended the use of both the PPVT-III-NL and Andringa et al.’s test when testing Dutch university students. According to them, these two tests allow a practical and broad assessment of vocabulary knowledge.

Andringa et al.’s receptive test consists of 60 multiple choice items. Participants are presented with written target words and are instructed the select one of 5 answer options. All target words are embedded in a neutral carrier phrase. The last answer alternative always displays; “*Ik weet het echt niet*.” [“I really don’t know”]. The other alternatives represent one correct and three incorrect definitions of the target word’s meaning.

**Figure 7:**
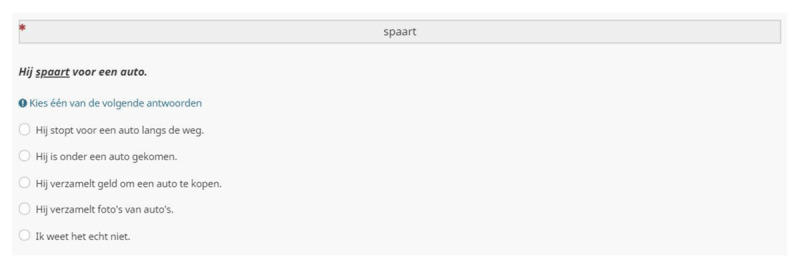
Receptive multiple choice test (Andringa et al., 2012); example item set-up.

We added the receptive multiple choice test to a copy of the PPVT/DAIVT questionnaire in Limesurvey. The test items were presented in the same order as was done by Andringa et al. (2012). ***Figure 7*** shows the item set-up.

### METHOD

#### Participants

A total of 270 participants were tested at this stage. They consisted of three samples, varying in age, education, and country of origin (Belgium [Flanders] or the Netherlands). All samples completed the PPVT-III-NL and the DAIVT. One sample also completed Andringa et al.’s test. All participants gave informed consent.

Because the validation happened during an exam period for university students, we reached out to two secondary schools and we were given permission to test pupils from the highest grades during a one-hour Dutch class. Only pupils who finished all tests within the one hour mark were included in the analysis (n = 52, 20 male and 32 female, ages 15–19, *M = 16.5; SD = 0.7*). The classes included some former OKAN students. The OKAN or *Onthaalonderwijs voor anderstalige nieuwkomers* program offers an intense training period of the Dutch language for non-Dutch speaking pupils.

University students were invited to participate in the online-based tests via private Ghent University groups on social media. To motivate participation, they had the opportunity to leave an e-mail address for a chance to win a cinema voucher. A random winner was picked and contacted after test administration. In all, 83 students completed the tests entirely (24 male, 59 female, ages 18–26; *M = 21.3; SD = 2.0*). The students were enrolled in varying degrees at Ghent University. Both bachelor and master students participated.

At the Max Planck Institute (MPI; Nijmegen, the Netherlands) participants were invited to complete several receptive vocabulary tests as part of ongoing research, two of which were the second version of the DAIVT and the PPVT-III-NL. In total, 135 native Dutch speakers finished both tests. Ages varied between 18 and 64 (*M = 34.8; SD = 15.8*). For these participants, a new version was made with a person from the Netherlands saying the target words.

#### Procedure

The order of tests was fixed for all participants. They all started with the PPVT-III-NL, followed by the second version of the DAIVT (104 test items and 2 practice items). The secondary school pupils also finished Andringa et al.’s test. All secondary school pupils and university students started with set 13 of the PPVT-III-NL (ages 16;0–35;11).

Due to technical difficulties in the schools’ pc-rooms, some secondary school pupils were forced to fill in the tests on their smartphone. Limesurvey allows completion of questionnaires on smartphones so this was not considered a problem. Most pupils had access to a personal computer. They were asked to bring their own headphones or earphones.

The university students were asked to fill in the PPVT and the DAIVT. Similar to the secondary school pupils, they accessed the tests via a link.

The researchers from the Max Planck Institute (MPI) were responsible for the PPVT-III-NL’s test administration (an online version). One week later, participants were sent an e-mail with a link to the DAIVT.

Total scores for the DAIVT and Andringa et al.’s test were shown after the test to reward participants for participation.

#### Analysis

Similar to the previous phase, we ran a reliability analysis for the DAIVT and a PCA for the individual test items. Furthermore, correlations between the three tests were calculated and PCAs were performed on the total vocabulary test scores.

### RESULTS

***Table 1*** displays the average vocabulary test scores with ranges for all three samples. The descriptive values show a noticeable positive shift from secondary school students to samples with older participants. This is true for both the PPVT and the DAIVT. As vocabulary size is known to increase with age (Keuleers, Stevens, Mandera, & Brysbaert, 2015; Schlichting, 2005), this is a first finding pointing to the validity of the test.

**Table 1:** Vocabulary test scores.


TEST SCORES				

*SECONDARY SCHOOL*	*N*	MINIMUM	MAXIMUM	MEAN*(SD)*

PPVT	52	147	190	169.4 *(8.4)*

DAIVT *(104 items)*	52	38	91	56.4 *(10.4)*

Andringa	52	20	45	35.5 *(6.0)*

***UGHENT STUDENTS***	***N***	**MINIMUM**	**MAXIMUM**	**MEAN*(SD)***

PPVT	83	162	198	182.7 *(6.5)*

DAIVT *(104 items)*	83	39	96	76.1 *(12.0)*

***MPI***	***N***	**MINIMUM**	**MAXIMUM**	**MEAN*(SD)***

PPVT	135	156	199	182.8 *(10.1)*

DAIVT *(104 items)*	135	46	101	80.1 *(12.9)*


#### Secondary school pupils

Among the secondary school participants, the DAIVT had a reliability of 0.81 (measured with Cronbach’s α). None of the test items were answered correctly by all pupils. Thirteen items (*triade, taboeret, metronoom, wasemen, beschroomd, glooiing, stuw, suède, tilde, kazoo, grimeren, schalks, and doceren*) correlated negatively with the total scale. A total of 27 test items had a factor loading of .30 or more.

The DAIVT correlated 0.55 with the PPVT *(p <.001)*, while Andringa et al.’s test correlated 0.44 with the PPVT *(p < .001)*. The correlation between the DAIVT and Andringa et al.’s test was 0.42 (*p < .01)*. A PCA for the vocabulary test scores revealed one component with an eigenvalue of >1. The component explained 64,8% of the total variance. The PPVT had the highest loading (*0.84*), closely followed by the DAIVT (*0.82*) and Andringa et al.’s test (*0.76*).

#### Ghent University students

The reliability measure Cronbach’s α was 0.89 for the sample of Ghent University students. All young adults selected the correct photo for the target words *baret* and *windturbine*. These items are considered too easy for this sample. Target words *flaneren, logistiek, soldeerbout, gevel, pastinaak, afgepeigerd, jutten, and doceren* had factor loadings below zero. On the other hand, 53 items had a loading of .30 or more.

Students’ DAIVT scores correlated 0.74 with the PPVT scores *(p < .001)*. The PCA with the DAIVT and PPVT test scores revealed one significant component (factor loading = *0.93*) with an explained variance of 87.2%.

#### Max Planck Institute participants

The reliability analysis with the MPI sample again indicated that the DAIVT is reliable (Cronbach’s α = 0.92). All MPI participants answered target words *stuw* and *luifel* correctly**. Only one test item *(stobbe)* had a negative factor loading and 57 test items had a loading of .30 or higher on the total scale. This is presumably a consequence of the larger age range in the sample.

The correlation between the DAIVT and the PPVT was 0.87 for the MPI participants *(p < .001)*. Again, the PCA with the vocabulary test scores presented one significant component (factor loading = *0.97*). The explained total variance was 93.4%.

#### Overall results

Altogether, the DAIVT performed well within all samples. The results reflected strong correlations between the PPVT-III-NL and the DAIVT. The total test score PCAs also revealed that most variance was explained by a single component, indicating the tests’ capability to measure a shared underlying ability.

Although significant, the test score correlations were less strong for secondary school participants. Given the pupils’ low average DAIVT score and the fewer items with a high factor loading, the current test version was considered particularly challenging for secondary school participants. A similar pattern was found for Andringa et al.’s test.

**Figure 8:**
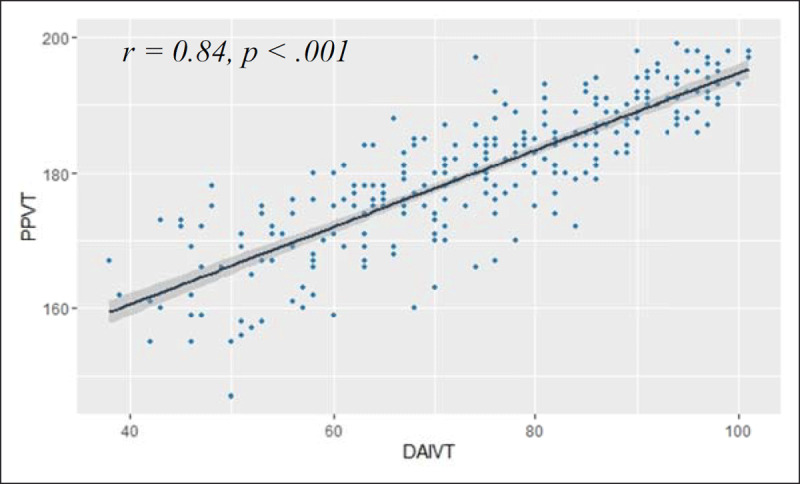
Correlation (and regression line with 95% confidence interval) between the DAIVT (second version with 104 items) and the PPVT-III-NL; cluster of 3 participant samples: 52 secondary school pupils, 83 university students, and 135 participants invited by the Max Planck Institute.

***Figure 8*** shows the strong correlation between the PPVT and the DAIVT of the combined samples with a 95% confidence interval regression line *(n = 270, r = .84, p < .001)*.

### DISCUSSION AND TEST ADJUSTMENTS

Convergent validity of the DAIVT’s second version was assessed with two receptive vocabulary tests, the established PPVT-III-NL and Andringa et al.’s receptive multiple choice vocabulary test. A total of three samples were included in the analyses. The PPVT-DAIVT correlation and explained test score variance was the highest in the sample with the largest age range (MPI participants). The lowest values, although still strong, were found for secondary school participants. The DAIVT performed well in the student sample, the target population of the study.

Again, the item-analysis pointed to test items that could use some revision. To improve the test, university students’ results were used as a reference. Simultaneously, we wanted to reduce the number of items in the DAIVT. Consequently, less performing items were removed.

In total, 14 test items *(taboeret, corsage, jutten, flaneren, nachtschadefamilie, sarcofaag, pastinaak, stobbe, soldeerbout, tilde, afgepeigerd, olfactorisch, windturbine*, and *doceren)* were removed from the test. Most of these items did not perform well for the second time (factor loading < .20), despite the item adjustments made in the previous phase.

Additionally, minor adjustments were made to some target and/or distractor images. Specifically items reflecting an attitude/emotion needed fine-tuning. After this phase, the DAIVT comprised 90 test items.

## PHASE III – VALIDITY (PART 2)

Finally, we tested the last version of the DAIVT with the target group for which the test was developed. Similarly to the previous phase, convergent validity was assessed.

### METHOD

#### Participants

A total of 50 first year psychology students from Ghent University participated in exchange for a credit to fulfill course requirements (5 male, 45 female). The students were recruited through Sona-Systems, a research participation system. Once they signed up for the study, they could access the tests via a link. The results from one female participant were removed from analysis because the age range of 17–26 y/o was exceeded. Ages varied between 17 and 22 (n = 49, *M = 18.6; SD = 0.9*).

At the Max Planck Institute an additional sample of Dutch participants from the MPI pool was invited to fill in the latest version of the DAIVT as part of an ongoing project. Results from 108 participants within the 17–26 y/o age range were included for analysis in the current study (47 male, 61 female, *M = 23.2; SD = 2.2*). All participants were or had been enrolled in higher education (24 in higher professional education (HBO), 84 in scientific education (WO)).

#### Procedure

All participants gave informed consent at the beginning of the test session. For the first year psychology student sample, the Limesurvey questionnaire included the PPVT-III-NL, the latest version of the DAIVT, and the receptive multiple choice test by Andringa et al. The order of test administration was fixed. The MPI participants completed the PPVT-III-NL and the DAIVT. All participants started with set 13 (ages 16;0–35;11) of the PPVT-III-NL. The DAIVT consisted of 90 test items. No practice items were added for the first year students. The latter was done because they already had experience with the PPVT. Again, participants were advised to use headphones or earphones. They could repeat the spoken word if needed. Total scores for the DAIVT and the receptive multiple choice vocabulary test (Andringa et al., 2012) were shown at the end of the respective test.

#### Analysis

The analyses were identical to the previous samples. We performed an item-analysis, assessed correlations between the tests, and used a PCA on the participants’ total vocabulary scores for the different tests.

### RESULTS

#### First year Ghent University student sample

***Table 2*** displays the descriptive values for the vocabulary test scores in the psychology student sample. Notice that the average test score is approximately 2/3 of the number of items in the test for both the DAIVT and Andringa et al.’s test. The mean test scores for the three tests indicate a similar difficulty.

**Table 2:** Vocabulary test scores.


*UNIVERSITY STUDENTS (1ST YEAR)*	*N*	MINIMUM	MAXIMUM	MEAN*(SD)*

PPVT	49	143	201	179.4 *(9.4)*

DAIVT *(90 items)*	49	28	88	58.5 *(12.2)*

Andringa (60 items)	49	25	55	39.5 *(6.0)*


The reliability measure Cronbach’s α suggested a good reliability for the DAIVT in the target population of first year students (α = 0.89). No-one had a perfect score and no test item was answered correctly by all students. The item-analysis revealed positive results. A considerable number of test items had a factor loading of .30 or higher (n = 46). In all, 3 items *(organogram, triade*, and *degressie)* correlated negatively with the total scale (see Appendix A). For 2 of these items, the negative loading was accounted for by a high correct:false ratio (46:3).

**Figure 9:**
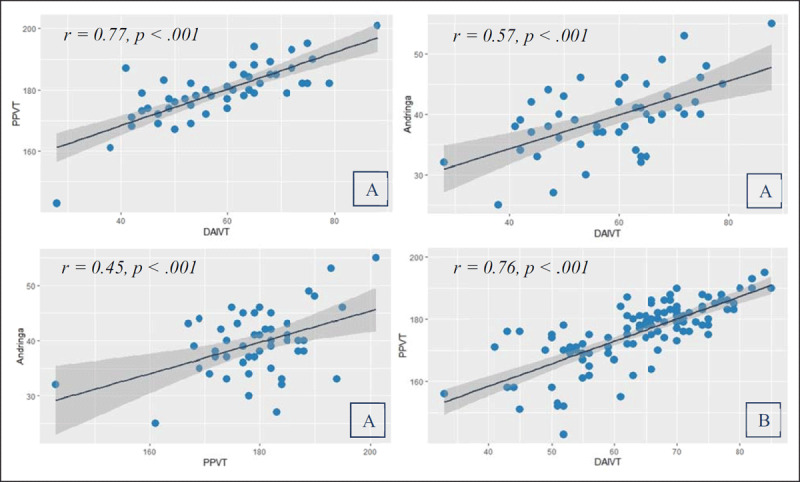
Correlations (and regression lines with 95% confidence interval) between the DAIVT (90 items), the PPVT-III-NL, and Andringa et al.’s test; **A** 49 first year psychology students, **B** 108 MPI participants (ages 18–26).

The strongest correlation was found between the PPVT and the DAIVT (*r = .77, p < .001*). The DAIVT and Andringa et al.’s test correlated less strongly (*r = .57, p <.001*) and the correlation between the PPVT and Andringa et al.’s test was moderate (*r = .45, p < .01*). ***Figure 9(A)*** reflects the correlations with their respective 95% confidence interval regression lines.

The PCA on the vocabulary scores for the three tests reflected only one component with an eigenvalue of >1. The component explained 73.5% of the total variance. As can be seen in ***Table 3***, the DAIVT had the highest factor score, followed by the PPVT and Andringa et al.’s test. When performing a PCA on only the PPVT and the DAIVT’s test scores, 88.6% of total variance was explained.

**Table 3:** PCA component loadings for all three tests.


	COMPONENT	UNIQUENESS

	1	

DAIVT	0.924	0.146

PPVT	0.876	0.233

Andringa	0.763	0.417


#### Max Planck Institute participants sample

***Table 4*** shows the descriptive values for the vocabulary test scores in the MPI participant sample. The average test score for the DAIVT was higher than the average score in the Ghent University sample. Given the higher average age of the MPI participants, we expected this. Surprisingly, the MPI participants’ PPVT average test score was lower than the average first year students’ score. Again, Cronbach’s α reflected a good reliability (α = 0.88). None of the test items were answered correctly by all test-takers and no-one had a flawless score. Altogether, 6 items *(baret, copieus, suède, degressie, tourniquet, and irrigatie)* had a negative factor loading while 45 items showed a high factor loading (≥.30) on the total scale. A high correct:false ratio explained the negative loadings for *baret* and *degressie*.

**Table 4:** Vocabulary test scores.


*MPI PARTICIPANTS*	*N*	MINIMUM	MAXIMUM	MEAN*(SD)*

PPVT	108	143	195	176.2 *(10.1)*

DAIVT *(90 items)*	108	33	85	64.6 *(10.6)*


The strength of the correlation between the DAIVT and the PPVT test scores was almost identical to the first year university sample (*r = .76, p < .001*). ***Figure 9(B)*** shows the scatter plot. The PCA with the test scores revealed one mutual component with a factor loading of *0.94*. The component explained 87.8% of the total variance.

### DISCUSSION

We tested our target population, which included both first year university students and other Dutch participants between the ages of 17 and 26. Again, the PPVT-III-NL, the latest version of the DAIVT (90 items), and Andringa et al.’s test were used as vocabulary measures. We provided descriptive and correlational values and performed a reliability analysis for the DAIVT and a PCA for the total vocabulary test scores.

We successfully improved the new test. The removal of test items did not appear to reflect a loss of information in the test scores. Overall, the DAIVT had good reliability and performed well in the current samples with young adults. The DAIVT correlated strongly with the PPVT. The correlation with Andringa et al.’s test was lower, indicating that the multiple choice written format diverges more (although this could also be due to the “don’t know” option).

## GENERAL DISCUSSION

Ideally, researchers interested in vocabulary knowledge have access to a wide range of vocabulary tests. This allows them to disentangle vocabulary knowledge from format-specific and test-specific factors. For instance, a researcher with access to a minimum of two tests with the PPVT format (spoken targets, picture selection) and a minimum of two tests with the Andringa et al. (2012) format (written targets, selection of written words) can determine to what extent both formats measure the same underlying latent variable (vocabulary knowledge) and to what extent they are influenced by format-specific factors. The latter works best when the tests were developed by different groups, because this reduces experimenter bias. By adding more tests, researchers can investigate to what extent test performance is affected in a systematic way by latent variables other than vocabulary knowledge. For instance, it could be that the Andringa et al. format, with its written presentation, is influenced more by reading pleasure than the PPVT format. This is the multitrait – multimethod (Campbell & Fiske, 1959).

The aim of the present study was to develop a new measure of receptive vocabulary size using the PPVT format (spoken targets, picture selection). We had several motivations to develop such a test. One was flexible use in research; another was to examine whether the format would still work if the line drawings were replaced by more easily available photographs. The target population was Dutch-speaking students in higher education (mostly university students), as this is the population in most of our studies.

We describe the construction of the test and how it was validated by comparing it to similar tests in the target population. The data show that the test works well, and possibly slightly better in the target population than PPVT-III-NL (Schlichting, 2005). In contrast, the latter seems to do slightly better for senior high-school students, in line with its broad remit. The new test is called the Dutch Auditory & Image Vocabulary Test (DAIVT). A comparison of PPVT and DAIVT with Andringa et al. (2012) provides evidence for format-specific factors, as both tests correlate less with Andringa et al. than with each other. Ideally, we could have included another test of the Andringa et al. format to make sure that these tests correlate more with each other as well than with PPVT and DAIVT. Such a test could be the one developed by Vander Beken et al. (2018).

The DAIVT comprises 90 test items and 1 practice item. The latter is the *stoel* [chair] item from the second phase. It is an item everyone with basic knowledge of Dutch should know and is used to illustrate the procedure. After completing it, the participant gets feedback about the correctness of their answer. The items are always administered in the same order and are self-paced. The DAIVT contains 53 nouns, 20 adjectives, and 17 verbs. Most target words can be subdivided in an array of semantic categories (***Table 5***). Although the items differ in difficulty (see Appendix A), they are not presented in terms of increasing difficulty, like in the PPVT-III-NL, because we advise always to run the complete test. It does not take longer than 10 minutes.

**Table 5:** The DAIVT’s 90 target words subdivided into 12 semantic categories and 2 additional lexical categories for verbs and adjectives without a pronounced semantic category. N = noun, V = verb, and A = adjective.


CATEGORY (SEMANTIC & LEXICAL)	90 TARGET WORDS

Related to the human body	*wervel (N), palperen (V), resuscitatie (N), laven (V), gesticuleren (V), grimeren (V), pectoraal (A)*

People	*koter (N), paleontoloog (N), estheet (N), centurion (N), couturier (N)*

Related to animals	*aalscholver (N), invertebraat (A), mammalogie (N), kokkel (N), kolonie (N)*

Related to food	*konfijten (V), savoureren (V), gastronomisch (A), confiserie (N), saffraan (N)*

Related to music	*metronoom (N), percussie (N), kazoo (N)*

Related to clothes and accessories	*baret (N), habijt (N), suède (N), ruiker (N)*

Furniture and tools	*plamuurmes (N), schuimspaan (N), hellebaard (N), zwachtel (N), divan (N)*

Related to nature, landscape or environment	*emissie (N), urbaan (A), uitbaggeren (V), glooiing (N), stuw (N), arctisch (A), pegel (N), halm (N), drasland (N), puimsteen (N), eruptie (N), calamiteit (N), irrigatie (N)*

Related to architecture or history	*gevel (N), arcade (N), boegbeeld (N), artefact (N), rozet (N), luifel (N), buste (N), prieel (N), erker (N), tourniquet (N)*

Related to transportation or money	*bolide (N), logistiek (N), konvooi (N), fiduciair (A), gebarricadeerd (V), intersectie (N)*

Emotion, attitude or behavior	*extatisch (A), sedentair (A), beschroomd (A), aversie (N), verbolgen (A), schalks (A)*

Abstract	*concentrisch (A), triade (N), gelobd (A), organogram (N), posterieur (A), lancetvormig (A), degressie (N)*

Additional verbs (actions)	*bivakkeren (V), buitelen (V), apporteren (V), wasemen (V), versmaden (V), confereren (V), bejubelen (V), kippen (V), keuvelen (V)*

Additional adjectives	*copieus (A), clandestien (A), nuptiaal (A), precair (A), gammel (A)*


The supplementary materials (available at *https://osf.io/8kxz7/files/*) contain all information for users who want to implement the test themselves, including information from the item analysis of the final version in case researchers are interested in these (see also Appendix A). We also provide percentile ranks (Appendix B), although we recommend to use the raw scores for research purposes. This provides the best information about the performance of a group of students in a particular study.

The test can be used freely for research purposes under the Creative Commons License *Attribution-NonCommercial-ShareAlike*. This means that others can remix, adapt, and build upon our work non-commercially, as long as they refer to the present article and license their new creations under identical, non-commercial terms as we do.

For researchers who simply want to have a test score of their participants, we also created an online version. Participants can choose to hear a Dutch Flemish voice or a Dutch voice from the Netherlands. Participants are asked to indicate their gender and age. They can also enter a code and email address you provide them with. If they do so, an email will be sent to the address. It will contain the total test score and the other information given by the participant, allowing you to include these in your analyses.

DAIVT is designed as a free and versatile test for research purposes, specifically aimed at students in higher education. It is not meant as a tool for clinical use in the general population. For such purpose, it is better to make use of an officially normed test such as PPVT-III-NL (Schlichting, 2005).

## Footnotes

The test can be reduced to 40 items without much loss of information (*http://crr.ugent.be/archives/2197*).

## SUPPLEMENTARY MATERIALS

Link to the DAIVT: *https://tpsurvey.ugent.be/limesurvey315/index.php/923234?lang=nl*.

Supplementary materials (word lists with photos and results) can be found on the Open Science Framework. *https://osf.io/8kxz7/*.

## ADDITIONAL FILES

The additional files for this article can be found as follows:

Appendix A. Percentage of individual test items answered correctly, factor loadings, and point-biserial correlations (rpb); FL = Flanders (first year university students), NL = the Netherlands (Dutch participants in higher education).

Appendix B. Percentile ranks of the samples’ total test scores; FL = Flanders (first year university students), NL = the Netherlands (Dutch participants in higher education).
