# Supplementary material for: The Dutch Auditory & Image Vocabulary Test (DAIVT): A New Dutch Receptive Vocabulary Test for Students
Source: Psychol Belg. 2021 Jan 19;61(1):1–17. doi: 10.5334/pb.552 (PMC7824982; doi:10.5334/pb.552)
Supplement: Appendix A. — Percentage of individual test items answered correctly, factor loadings, and point-biserial correlations (rpb); FL = Flanders (first year university students), NL = the Netherlands (Dutch participants in higher education). [file pb-61-1-552-s1.pdf]

**Appendix A:** Percentage of individual test items answered correctly, factor loadings, and point-biserial correlations ($r_{pb}$); FL= Flanders (first year university students), NL= the Netherlands (Dutch participants in higher education)

| Test item | % Answered correctly | | Factor loadings | | $r_{pb}$ *(p-value)* | |
|---|---|---|---|---|---|---|
| | *FL* | *NL* | *FL* | *NL* | *FL* | *NL* |
| *1. baret* | 94 | 99 | .18 | -.05 | .14 *(p= .324)* | -.06 *(p= .548)* |
| *2. gevel* | 86 | 87 | .43 | .40 | .40 *(p= .005)* | .36 *(p< .001)* |
| *3. koter* | 61 | 94 | .38 | .41 | .38 *(p= .006)* | .34 *(p< .001)* |
| *4. aalscholver* | 63 | 74 | .30 | .30 | .28 *(p= .056)* | .31 *(p= .001)* |
| *5. bivakkeren* | 61 | 94 | .43 | .23 | .39 *(p= .006)* | .23 *(p= .018)* |
| *6. extatisch* | 86 | 94 | .44 | .33 | .42 *(p= .003)* | .31 *(p< .001)* |
| *7. concentrisch* | 88 | 85 | .09 | .04 | .14 *(p= .325)* | .07 *(p= .485)* |
| *8. triade* | 94 | 95 | -.10 | .07 | -.05 *(p= .718)* | .06 *(p= .521)* |
| *9. buitelen* | 24 | 49 | .55 | .62 | .56 *(p< .001)* | .62 *(p< .001)* |
| *10. wervel* | 86 | 89 | .26 | .19 | .22 *(p= .123)* | .18 *(p= .056)* |
| *11. bolide* | 37 | 75 | .53 | .56 | .57 *(p< .001)* | .51 *(p< .001)* |
| *12. paleontoloog* | 82 | 93 | .03 | .01 | .04 *(p= .803)* | .03 *(p= .763)* |
| *13. gelobd* | 84 | 76 | .14 | .39 | .16 *(p= .289)* | .38 *(p< .001)* |
| *14. sedentair* | 61 | 78 | .20 | .19 | .22 *(p= .137)* | .20 *(p= .037)* |
| *15. organogram* | 69 | 80 | -.12 | .13 | -.07 *(p= .642)* | .13 *(p= .193)* |
| *16. konfijten* | 88 | 94 | .43 | .11 | .40 *(p= .004)* | .12 *(p= .224)* |
| *17. apporteren* | 92 | 82 | .46 | .33 | .42 *(p= .002)* | .36 *(p< .001)* |
| *18. emissie* | 73 | 81 | .10 | .36 | .12 *(p= .413)* | .34 *(p< .001)* |
| *19. plamuurmes* | 92 | 94 | .60 | .34 | .52 *(p< .001)* | .32 *(p< .001)* |

| | | | | | | |
|---|---|---|---|---|---|---|
| *20. copieus* | 51 | 45 | .27 | -.09 | .31 *(p= .031)* | -.01 *(p= .921)* |
| *21. metronoom* | 76 | 74 | .15 | .18 | .14 *(p= .336)* | .21 *(p= .032)* |
| *22. wasemen* | 35 | 41 | .30 | .40 | .32 *(p= .025)* | .38 *(p< .001)* |
| *23. logistiek* | 59 | 99 | .02 | .14 | .05 *(p= .712)* | .12 *(p= .236)* |
| *24. habijt* | 53 | 53 | .36 | .37 | .40 *(p= .005)* | .40 *(p< .001)* |
| *25. percussie* | 78 | 82 | .36 | .56 | .38 *(p= .007)* | .54 *(p< .001)* |
| *26. savoureren* | 43 | 18 | .18 | .22 | .22 *(p= .128)* | .25 *(p= .008)* |
| *27. urbaan* | 82 | 96 | .36 | .15 | .32 *(p= .023)* | .17 *(p= .081)* |
| *28. estheet* | 82 | 74 | .23 | .53 | .23 *(p= .115)* | .53 *(p< .001)* |
| *29. schuimspaan* | 71 | 73 | .21 | .25 | .21 *(p= .151)* | .24 *(p= .013)* |
| *30. uitbaggeren* | 53 | 87 | .35 | .42 | .36 *(p= .011)* | .38 *(p< .001)* |
| *31. boegbeeld* | 47 | 78 | .39 | .55 | .40 *(p= .004)* | .54 *(p< .001)* |
| *32. gastronomisch* | 92 | 76 | .48 | .54 | .45 *(p= .001)* | .54 *(p< .001)* |
| *33. palperen* | 45 | 66 | .33 | .19 | .32 *(p= .024)* | .24 *(p= .012)* |
| *34. arcade* | 71 | 67 | .29 | .27 | .29 *(p= .046)* | .30 *(p= .001)* |
| *35. artefact* | 78 | 86 | .46 | .22 | .43 *(p= .002)* | .22 *(p= .019)* |
| *36. posterieur* | 82 | 77 | .28 | .23 | .30 *(p= .038)* | .24 *(p= .013)* |
| *37. hellebaard* | 47 | 49 | .53 | .09 | .51 *(p< .001)* | .09 *(p= .367)* |
| *38. clandestien* | 63 | 42 | .28 | .11 | .28 *(p= .049)* | .15 *(p= .111)* |
| *39. lancetvormig* | 67 | 83 | .36 | .39 | .38 *(p= .008)* | .36 *(p< .001)* |
| *40. beschroomd* | 57 | 61 | .40 | .34 | .40 *(p= .006)* | .35 *(p< .001)* |
| *41. rozet* | 69 | 55 | .21 | .07 | .22 *(p= .122)* | .13 *(p= .166)* |
| *42. versmaden* | 43 | 44 | .25 | .29 | .30 *(p= .040)* | .31 *(p= .001)* |
| *43. nuptiaal* | 35 | 25 | .32 | .25 | .36 *(p= .012)* | .32 *(p< .001)* |
| *44. stuw* | 88 | 97 | .26 | .15 | .25 *(p= .088)* | .15 *(p= .114)* |

| | | | | | | |
|---|---|---|---|---|---|---|
| 45. *glooiing* | 37 | 39 | .14 | .36 | .19 *(p= .185)* | .38 *(p< .001)* |
| 46. *confereren* | 80 | 81 | .25 | .20 | .23 *(p= .106)* | .20 *(p= .038)* |
| 47. *arctisch* | 80 | 83 | .61 | .53 | .56 *(p< .001)* | .51 *(p< .001)* |
| 48. *luifel* | 94 | 97 | .19 | .29 | .17 *(p= .238)* | .26 *(p= .007)* |
| 49. *aversie* | 86 | 94 | .12 | .45 | .15 *(p= .300)* | .37 *(p< .001)* |
| 50. *buste* | 84 | 73 | .65 | .60 | .56 *(p< .001)* | .57 *(p< .001)* |
| 51. *bejubelen* | 49 | 84 | .36 | .38 | .38 *(p= .007)* | .41 *(p< .001)* |
| 52. *prieel* | 61 | 83 | .50 | .59 | .49 *(p< .001)* | .54 *(p< .001)* |
| 53. *resuscitatie* | 39 | 51 | .38 | .23 | .41 *(p= .003)* | .26 *(p= .006)* |
| 54. *halm* | 31 | 61 | .34 | .59 | .38 *(p= .008)* | .56 *(p< .001)* |
| 55. *kippen* | 92 | 95 | .10 | .00 | .07 *(p= .645)* | .02 *(p= .831)* |
| 56. *pegel* | 59 | 86 | .38 | .25 | .38 *(p= .007)* | .27 *(p= .005)* |
| 57. *zwachtel* | 47 | 79 | .50 | .48 | .52 *(p< .001)* | .46 *(p< .001)* |
| 58. *drasland* | 80 | 94 | .29 | .42 | .25 *(p= .078)* | .38 *(p< .001)* |
| 59. *invertebraat* | 61 | 60 | .41 | .36 | .41 *(p= .003)* | .39 *(p< .001)* |
| 60. *suède* | 63 | 82 | .05 | -.15 | .06 *(p= .671)* | -.08 *(p= .428)* |
| 61. *erker* | 76 | 87 | .53 | .44 | .52 *(p< .001)* | .42 *(p< .001)* |
| 62. *confiserie* | 33 | 22 | .31 | .20 | .34 *(p= .017)* | .23 *(p= .019)* |
| 63. *laven* | 49 | 24 | .40 | .27 | .40 *(p= .005)* | .31 *(p= .001)* |
| 64. *degressie* | 94 | 97 | -.09 | -.08 | -.05 *(p= .754)* | -.08 *(p= .406)* |
| 65. *saffraan* | 69 | 85 | .37 | .23 | .37 *(p= .008)* | .25 *(p= .010)* |
| 66. *precair* | 33 | 32 | .10 | .26 | .15 *(p= .311)* | .29 *(p= .003)* |
| 67. *mammalogie* | 39 | 70 | .44 | .46 | .44 *(p= .001)* | .44 *(p< .001)* |
| 68. *gesticuleren* | 69 | 61 | .22 | .31 | .21 *(p= .141)* | .32 *(p< .001)* |
| 69. *kokkel* | 59 | 55 | .31 | .26 | .30 *(p= .038)* | .26 *(p= .008)* |

| | | | | | | |
|---|---|---|---|---|---|---|
| *70. centurion* | 53 | 67 | .53 | .34 | .54 *(p< .001)* | .36 *(p< .001)* |
| *71. kolonie* | 51 | 73 | .28 | .25 | .32 *(p= .025)* | .26 *(p= .007)* |
| *72. konvooi* | 69 | 90 | .61 | .53 | .54 *(p< .001)* | .49 *(p< .001)* |
| *73. tourniquet* | 61 | 41 | .08 | -.11 | .10 *(p= .489)* | -.06 *(p= .553)* |
| *74. gammel* | 92 | 98 | .22 | .43 | .21 *(p= .150)* | .36 *(p< .001)* |
| *75. kazoo* | 67 | 49 | .44 | .25 | .43 *(p= .002)* | .28 *(p= .003)* |
| *76. fiduciair* | 35 | 30 | .46 | .15 | .45 *(p= .001)* | .18 *(p= .058)* |
| *77. puimsteen* | 65 | 76 | .20 | .42 | .23 *(p= .110)* | .42 *(p< .001)* |
| *78. eruptie* | 76 | 93 | .12 | .09 | .15 *(p= .309)* | .10 *(p= .306)* |
| *79. couturier* | 90 | 68 | .39 | .52 | .36 *(p= .010)* | .55 *(p< .001)* |
| *80. verbolgen* | 35 | 40 | .20 | .48 | .26 *(p= .071)* | .49 *(p< .001)* |
| *81. calamiteit* | 39 | 93 | .24 | .39 | .29 *(p= .047)* | .36 *(p< .001)* |
| *82. gebarricadeerd* | 90 | 97 | .20 | .30 | .19 *(p= .187)* | .25 *(p= .008)* |
| *83. grimeren* | 71 | 76 | .43 | .39 | .38 *(p= .007)* | .39 *(p< .001)* |
| *84. divan* | 49 | 56 | .37 | .53 | .37 *(p= .009)* | .55 *(p< .001)* |
| *85. schalks* | 35 | 43 | .38 | .24 | .38 *(p= .008)* | .27 *(p= .005)* |
| *86. keuvelen* | 59 | 66 | .19 | .51 | .20 *(p= .158)* | .49 *(p< .001)* |
| *87. pectoraal* | 65 | 69 | .27 | .11 | .26 *(p= .068)* | .14 *(p= .148)* |
| *88. intersectie* | 73 | 82 | .57 | .30 | .54 *(p< .001)* | .31 *(p= .001)* |
| *89. ruiker* | 41 | 28 | .24 | .08 | .28 *(p= .053)* | .08 *(p= .423)* |
| *90. irrigatie* | 86 | 91 | .03 | .43 | .07 *(p= .612)* | .41 *(p< .001)* |
