# Supplementary material for: The Dutch Auditory & Image Vocabulary Test (DAIVT): A New Dutch Receptive Vocabulary Test for Students
Source: Psychol Belg. 2021 Jan 19;61(1):1–17. doi: 10.5334/pb.552 (PMC7824982; doi:10.5334/pb.552)
Supplement: Appendix B. — Percentile ranks of the samples’ total test scores; FL = Flanders (first year university students), NL = the Netherlands (Dutch participants in higher education). [file pb-61-1-552-s2.pdf]

Appendix B: Percentile ranks of the samples' total test scores; FL= Flanders (first year university students), NL= the Netherlands (Dutch participants in higher education)

Percentiles	Score	
	FL	*NL*
0	28.00	33.00
1	32.80	41.14
2	37.60	43.00
3	39.32	43.21
4	40.76	44.28
5	41.40	45.00
6	41.88	46.68
7	42.00	49.49
8	42.00	50.00
9	42.64	50.00
10	43.60	50.70
11	44.00	51.00
12	44.00	51.84
13	44.24	52.00
14	44.72	52.00
15	45.40	52.05
16	46.36	53.00
17	47.00	53.19
18	47.00	54.00

19	47.12	54.33
20	47.60	55.00
21	48.08	55.00
22	48.56	55.00
23	49.00	55.61
24	49.00	56.00
25	49.00	56.00
26	49.48	56.00
27	49.96	58.67
28	50.00	59.00
29	50.00	59.03
30	50.80	60.10
31	51.76	61.00
32	52.36	61.24
33	52.84	62.00
34	53.00	62.00
35	53.00	62.00
36	53.00	62.00
37	53.00	62.59
38	53.24	63.00
39	53.72	63.00
40	54.40	63.80
41	55.36	64.00
42	56.00	64.00

43	56.00	64.01
44	56.12	65.00
45	56.60	65.00
46	57.24	65.00
47	58.68	65.29
48	60.00	66.00
49	60.00	66.00
50	60.00	66.00
51	60.00	66.00
52	60.00	66.00
53	60.44	66.71
54	60.92	67.00
55	61.00	67.00
56	61.00	67.00
57	61.72	67.99
58	62.68	68.00
59	63.00	68.00
60	63.00	68.20
61	63.28	69.00
62	63.76	69.00
63	64.00	69.41
64	64.00	70.00
65	64.00	70.00
66	64.00	70.00

67	64.16	70.00
68	64.64	70.00
69	65.00	70.00
70	65.00	70.00
71	65.00	70.97
72	65.00	71.00
73	65.04	71.00
74	65.52	71.18
75	66.00	72.00
76	66.96	72.00
77	67.92	72.39
78	68.00	73.46
79	68.00	74.00
80	68.40	74.00
81	68.88	74.00
82	69.72	74.00
83	70.68	74.81
84	71.32	75.00
85	71.80	75.00
86	72.00	75.02
87	72.00	76.09
88	72.48	77.00
89	73.44	77.00
90	74.20	77.00

91	74.68	77.37
92	75.00	78.00
93	75.00	78.51
94	75.12	79.00
95	75.60	79.65
96	76.24	81.44
97	77.68	82.00
98	79.36	83.72
99	83.68	84.93
100	88.00	85.00
